# CMV endotheliitis: a cause for recurrent failed corneal transplant

**DOI:** 10.3205/oc000082

**Published:** 2017-12-22

**Authors:** Nurul Ain Shahrudin, Aida Zairani Mohd Zahidin, Umi Kalthum Md Noh, Wan Haslina Wan Abdul Halim, Norshamsiah Md Din

**Affiliations:** 1 Pusat Perubatan Universiti Kebangsaan Malaysia, Cheras, Kuala Lumpur, Malaysia

**Keywords:** cytomegalovirus, endotheliitis, penetrating keratoplasty, corneal endothelium

## Abstract

**Objective:** To highlight the clinical presentation of CMV endotheliitis and the challenge in diagnosing this condition in recurrent failed penetrating keratoplasty (PK).

**Methods:** Case series

**Results:** There are 3 cases of recurrent failure in PK secondary to CMV endotheliitis presented. Case 1 and 2 were pseudophakic patients, while in case 3, the patient had a previous history of recurrent anterior uveitis. Case 1 and 3 had four and one previous failed PK respectively, while case 2 had endothelial keratoplasty twice before the diagnosis of CMV endotheliitis was made, following positive culture of aqueous humour. The visual acuity ranged from 1/60 to hand movement. All patients had pigmented KP, and two of them had typical coin-shaped KP. Oral valganciclovir was instituted for all patients consisting of 900 mg bidaily for two weeks, followed by 900 mg once daily for six months. Additionally, topical ganciclovir eyedrop 0.5% was given every four hours with topical dexaminim four times a day. Repeated anterior chamber (AC) tap after six months of treatment was negative for CMV in case 3 while cases 1 and 2 are still on treatment. CMV endotheliitis is an increasingly important cause of failed corneal transplant. We recommend anterior chamber tap in suspicious cases of repeatedly failed corneal transplant, regardless of the presence of coin-shaped KP or not. Minimum treatment with oral valganciclovir is important to eradicate the problem, before proceeding with another corneal transplant.

**Conclusion:** It is important to make an accurate early diagnosis by good clinical judgement in preventing loss of corneal endothelial cells. High index of suspicion for CMV endotheliitis as a cause of graft failure must be made especially when the patient presents with coin-shaped KP. Therefore unnecessary treatment resulting from misdiagnosis in these patients can be prevented. Early recognition and treatment of this condition is important to prevent permanent endothelial cell loss and corneal decompensation.

## Introduction

Cytomegalovirus (CMV) related corneal endotheliitis is an inflammation of the corneal endothelium caused by CMV. It typically presents as coin-shaped keratic precipitates (KP), with or without corneal edema in immunocompetent individuals [1]. This case series highlight the clinical presentation of CMV endotheliitis and the challenge in diagnosing this condition in recurrent failed penetrating keratoplasty (PK).

## Case descriptions

### Case 1 

A 71-year-old Malay man with a history of bilateral corneal laser refractive surgery and bilateral cataract surgery done at private centre complicated with cornea decompensation and multiple penetrating keratoplasty. Both eyes developed advanced steroid induced glaucoma. Right eye Ahmad valve tube and transcleral cyclophotocoagulation was done and visual acuity was counting finger postoperatively. Left eye visual acuity was 1/60 post Baerveldt tube implantation. Postoperatively his left eye vision improved to 2/60. He developed left eye cornea decompensation after one year and re-PK was done. 

**Figure 1:**
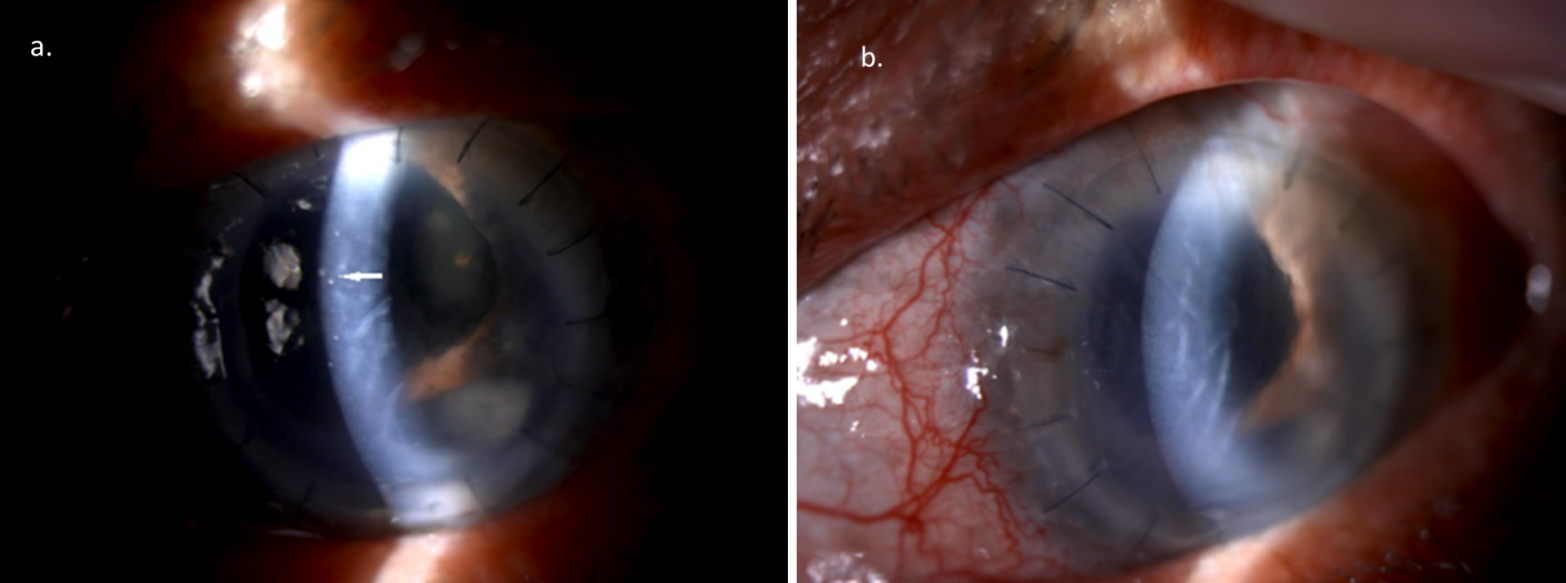
a. Coin-shaped keratic precipitate on the cornea endothelium (white arrow). b. The keratic precipitate resolved after starting treatment.

His left eye vision deteriorated to 1/60 after three months of re-PK and developed early graft rejection. The left eye had hazy cornea with bullae, descemet fold, coin-shaped pigmented keratic precipitate, but the anterior chamber (AC) was deep and quiet. The intraocular pressure (IOP) was 12 mmHg. There was no khodadoust line and no fundus view (Figure 1a (Fig. 1)).

The patient was initially started on intravenous methylprednisolone 500 mg daily for three days followed by tapering dose of oral prednisolone alongside dexaminim eyedrop. Left eye AC tap done showed positive for CMV PCR. HSV and VZV PCR from the aqueous sample were negative. Serum CMV IgM and IgG were not taken. Therefore, he was treated as CMV endotheliitis. He was started on oral valganciclovir 900 mg bidaily for two weeks followed by 900 mg daily for six months and ganciclovir eyedrop 0.5% four hourly left eye. His left eye visual acuity improved to 3/60 and coin-shaped KP disappeared (Figure 1b (Fig. 1)) after three weeks of treatment. He was planned for repeated AC tap after six months of treatment and was considered to undergo re-PK.

### Case 2

A 70-year-old Chinese man with bilateral Fuchs’ endothelial dystrophy, bilateral pseudophakic and right eye complicated with posterior capsule rent and drop cortical matter about two years ago. The best corrected right eye visual acuity was 6/12 postoperatively. 

**Figure 2:**
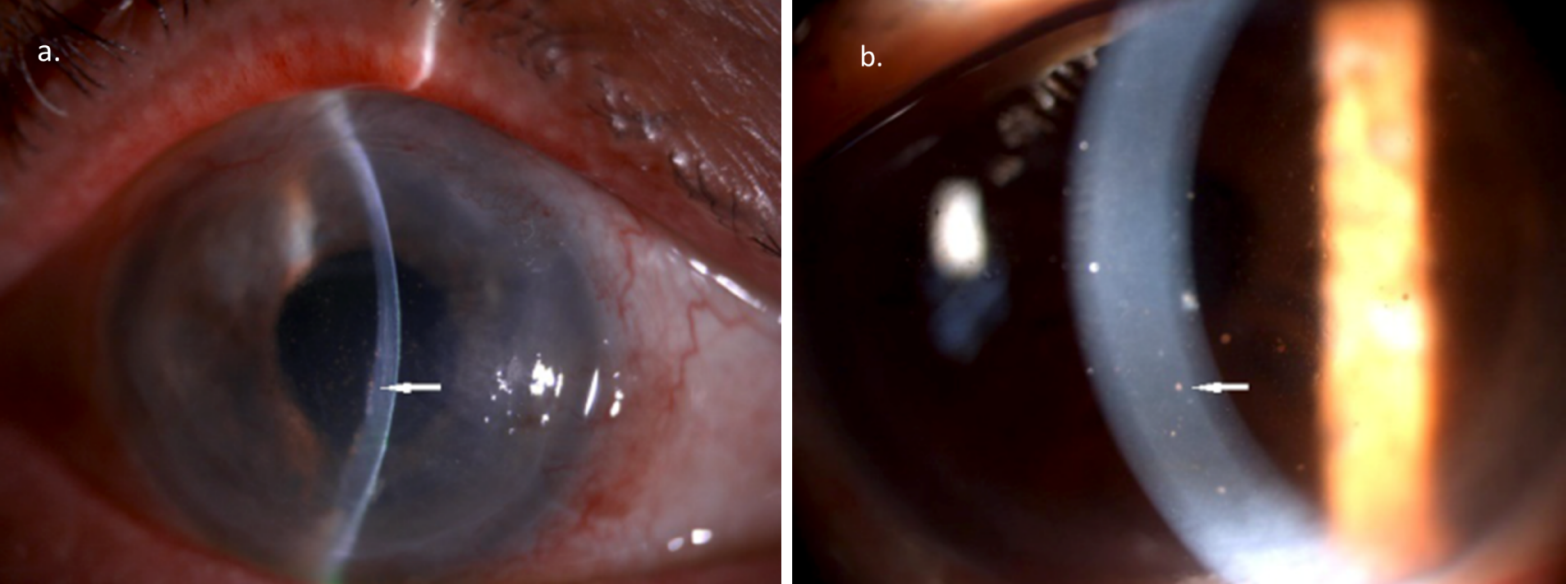
a. Coin-shaped keratic precipitate on the cornea endothelium (white arrow). b. The keratic precipitate (white arrow) reduced after starting on treatment.

His right eye vision dropped to 6/36 at 3 months postoperatively. He was noted to develop cornea decompensation and diagnosed as pseudophakic bullous keratopathy. The cornea was hazy with epithelial bedewing, descemet striae and coin-shaped pigmented KP on cornea endothelium (Figure 2a (Fig. 2)).

Right eye descemet stripping automated endothelial keratoplasty (DSAEK) was done. He developed steroid induced glaucoma and underwent right eye trabeculectomy with mitomycin C 0.02%. About one week after that, right eye visual acuity worsened to 4/60 due to early graft rejection. Intravenous methylprednisolone 500 mg daily was instituted for three days alongwith topical dexaminim hourly followed by oral prednisolone with tapering dose. However right eye visual acuity further worsened to 1/60. Right eye re-DSAEK was done for graft failure. The second cycle of intravenous methylprednisolone 500 mg daily was given. Aqueous sampling done was positive for CMV. HSV and VZV PCR from the aqueous sample were negative. Serum CMV IgM and IgG were not taken. He was started on oral valganciclovir 900 mg bidaily for two weeks and 900 mg daily for six months. Visual acuity improved to 2/60 after two weeks of treatment and KP reduced (Figure 2b (Fig. 2)). Repeated AC tap will be done in six months time and re-PK considered.

### Case 3

A 56-year-old Chinese man developed left eye decompensated cornea following recurrent anterior uveitis. He also developed left eye secondary glaucoma with intraocular pressure 28 mmHg due to long-term steroid use and left eye Baerveldt tube was done. Initial right eye visual acuity was 6/6, while left eye was 6/24. 

PK was done twice in another center. Examination revealed hazy cornea with descemet fold and diffuse pigmented KP. Aqueous tap was positive for CMV PCR. HSV and VZV PCR from the aqueous sample were negative. Serum CMV IgM and IgG were not taken. He was treated as CMV endotheliitis. 

Oral valganciclovir 900 mg bidaily was started for two weeks followed by 900 mg daily completed for six months alongwith topical dexaminim hourly left eye and tapered gradually. Repeated AC tap after six months showed negative CMV result. However, his vision worsened to hand movement due to cornea decompensation. Left eye re-PK was done three months after he completed oral valganciclovir. Visual acuity post PK improved to 6/60 after two months. The patient continued his follow-up elsewhere. 

## Discussion

Cornea graft rejection and viral infection can present with very similar clinical presentation, particularly in cases with multiple history of penetrating keratoplasty. It could be the cause of early rejection as reported in this case series. Recent research has found that viruses like CMV, HSV and VZV could be the culprit of multiple cornea graft failure. As in this case, CMV can cause endothelial inflammation and promote failure of the cornea graft [1].

Rae et al. reported a case of CMV endotheliitis after penetrating keratoplasty presenting with diffuse keratic pigmentation and confirmed by a positive aqueous sampling for CMV PCR. The keratic pigmentation disappeared after starting on CMV treatment [2]. Soon et al. also reported a case series of CMV endotheliitis among immunocompetent Chinese patients. However, the cases were not associated with recurrent failed PK and KP had variable appearances [3]. Our case series concur with these previous case series with a positive aqueous sampling for CMV.

In CMV infection, KP is typically arranged in a coin-shaped pattern at the edge of the corneal edema during the disease progress, furthermore mild anterior chamber reaction and corneal oedema resulting from corneal endothelium destruction. Noriko et al. have found that coin-shaped lesion KP and corneal oedema were observed in 70.6% and 73.4% of the eyes [4]. In cornea graft rejection, clinical presentation might be similar in which graft edema, keratic precipitates, and anterior chamber reaction may also be present but khodadoust line is typical for graft rejection [5]. Corneal endotheliitis is a clinical diagnosis and the presence of coin-shaped KP could be used as a screening tool for CMV-related anterior segment infection, especially if it is associated with high IOP and corticosteroid-recalcitrant inflammation. In cases of CMV-related corneal endotheliitis, isolation of the virus from the anterior chamber is necessary before starting the required treatment [6].

The mechanism of corneal endotheliitis is not clear. All the corneal graft was screened and negative for CMV, VZV, HSV prior to the surgery. However, autoimmune process plays an important role. Suzuki and Ohashi have found that anterior chamber-associated immune deviation (ACAID) is being stimulated during intermittent reactivation of the virus [7]. Infection will occur in the eye when the antibodies are unable to attack the reactivated virus that is being transmitted via either one of the cornea transplants. Research by Bale et al. has shown that immunocompetent mice get anterior segment CMV infection, with only minimal involvement of the posterior segment [8]. These findings are similar in our cases in which anterior segment infection with CMV endotheliitis cases occur in immunocompetent patients while a more severe CMV infection involving the posterior segment will occur in immunosuppressed patients. The prolonged usage of steroid in patients with post PK could precipitate the onset of infection and stimulation of ACAID.

The treatment of CMV-related corneal endotheliitis should cover both infectious and inflammatory components. Systemic antiviral treatment with topical corticosteroids should be started. Ganciclovir is a potent antiviral medication used to treat CMV infections by arresting viral replication [9].

## Conclusion

It is important to make an accurate early diagnosis by good clinical judgement in preventing loss of corneal endothelial cells. High index of suspicion for CMV endotheliitis as a cause of graft failure must be made especially when the patient presents with coin-shaped KP. Therefore, unnecessary treatment resulting from misdiagnosis in these patients can be prevented. Early recognition and treatment of this condition is important to prevent permanent endothelial cell loss and corneal decompensation.

## Notes

### Competing interests

The authors declare that they have no competing interests.
